# Liposomes with an Ethanol Fraction as an Application for Drug Delivery

**DOI:** 10.3390/ijms19123806

**Published:** 2018-11-29

**Authors:** Ewa Pilch, Witold Musiał

**Affiliations:** Department of Physical Chemistry, Pharmaceutical Faculty, Wroclaw Medical University, Borowska 211, Wroclaw 50-556, Poland; ewa.pilch@student.umed.wroc.pl

**Keywords:** liposomes, ethosomes, ethanol, drug delivery, stability, electrokinetic potential

## Abstract

Liposomes containing a certain amount of ethanol are often referred to in the literature as ethosomes. Liposomes vary in size from 25 nm to 25,000 nm. Ethosomes are defined as lipids composed of phospholipids, ethanol, or isopropyl alcohol in quite high concentrations, and water. They enable active substances to reach the deep skin layers or even the general circulation. The structure of ethosomes allows for an increased penetration of the drug through two effects: the ethanolic effect and the ethosomal effect. Ethosomes can be obtained using three methods: the hot method, the cold method, and the classic method of mechanical dispersion. The literature describes many of the therapeutic groups of drugs that can be enclosed in ethosomal formulations. These include anti-viral drugs, antineoplastic drugs, antifungal drugs, hypnotic drugs, hormones, and many others. Ethosomes show important practical advantages compared to classic liposomes. It is necessary to conduct research with regard to real pharmaceutical systems using advanced physicochemical techniques.

## 1. Introduction

**Figure 1:**
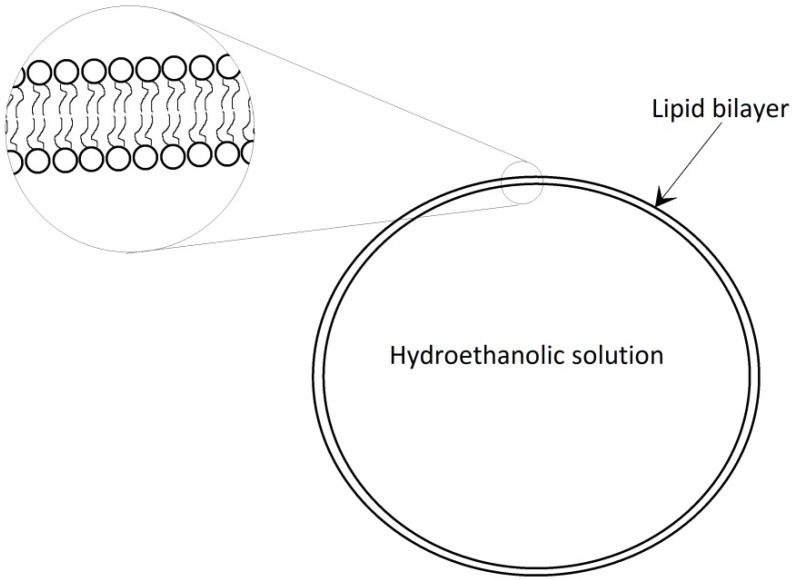
The structure of ethosomes.

**Figure 2:**
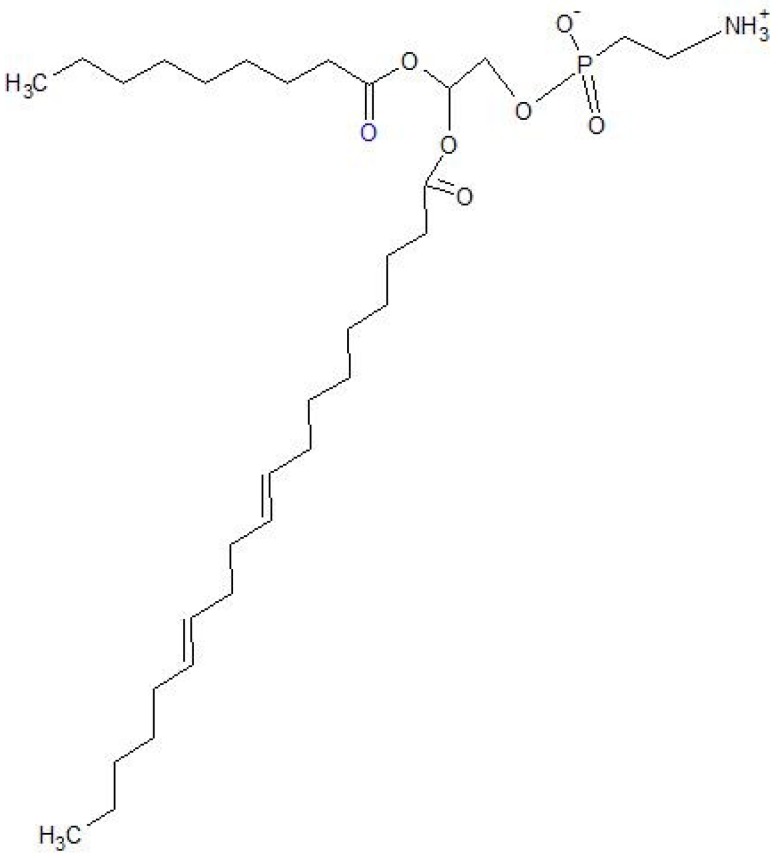
Soy lecithin—chemical structure.

Liposomes containing a certain amount of ethanol are often referred to in the literature as ethosomes (Figure 1). The size of liposomes varies between 25 nm and 25,000 nm [1]. Ethosomes are defined as lipid drug carriers composed of phospholipids, ethanol, or isopropyl alcohol in quite high concentrations, and water [2]. Ethosomes are composed of phospholipids, lower aliphatic alcohols containing from two to four carbon atoms, water, and an active ingredient. Instead of the alcohol, a mixture of ethanol and propylene glycol may be used. The alcohol content varies from 20% to 50%. The content of a mixture of propylene glycol and alcohol can be up to 70%. The phospholipids that build the ethosomes can be phosphatidylcholine (PC), hydrogenated phosphatidylcholine, phosphatidylserine (PS), phosphatidylinositol (PI), and others [3]. The ideal phospholipid should spontaneously and stably form a lipid bilayer in water [4]. In the human body, phospholipids are present in every cell. Sphingolipids are mainly found in the digestive system. They perform a structural function, affect the absorption of substances, and can also act as receptors [5]. PS acts as a neurotransmitter and is a building block of nerve cells. Cholesterol in cell membranes facilitates the organization of lipids and is a precursor for steroid biosynthesis in the nervous system [6]. Every change in the organization of lipids affects the metabolism and functioning of cells [7]. The lipids found in human skin are ceramides, cholesterol, cholesterol esters, and fatty acids in varying proportions. They are a barrier preventing the penetration of external factors into the internal environment of the organism [8]. Reducing the amount of individual lipids in the skin can affect the development of skin diseases. In atopic dermatitis, a decrease in the concentration of phosphatidylethanolamine is observed [9]. Phospholipids, such as soy lecithin (Figure 2) or egg lecithin, are most commonly used in the industry [10]. Alcohol is used in semi-solid emulsion systems as a penetration enhancer [11], and propylene glycol as a humectant [12]. Ethosomal carriers containing 30% ethanol were more stable and characterized by a good binding ability to the active substance according to studies conducted by Chandran [13]. However, the topical use of ethanol may result in adverse effects [14]. It may cause damage to the skin and the mucosal surface. Ethanol also has a carcinogenic potential in long-term applications to the mucous membranes [15]. It may influence skin microcirculation and functional capillary density [16].

## 2. Mechanism of Action

Ethosomes are carriers for medicinal substances administered to the skin and appendages of the skin. They enable the active substance to reach the deep skin layers or even the general circulation. The structure of ethosomes allows for an increased penetration of the drug through two effects: the ethanolic effect and the ethosomal effect [17]. The ability to penetrate the active substance through the epidermal barrier can be described by Fick’s first law [18]. Fick’s first law describes diffusion as a spontaneous and irreversible process leading to the equalization of a substance’s concentration [19]. It is expressed by the formula

(1) J=−DΔC

where *J* is the particle diffusion stream, *D* is the diffusion coefficient, and Δ*c* is the difference in concentration [20].

**Figure 3:**
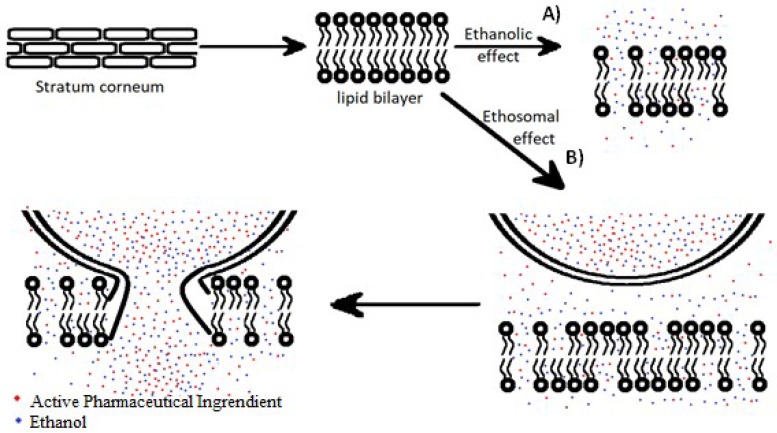
(**A**) The ethanolic and (**B**) ethosomal effects of ethosomes.

The quantity of ethanol contained in ethosomes is a known factor which increases the penetration of the active substances. Higher concentrations of ethanol in ethosomes make the penetration of the drug into the deeper layers of the skin more effective than in the use of classical liposomes or water–alcohol solutions [21]. Ethanol increases the fluidity of the lipid membrane and reduces the density of lipids in the cell membrane. The partial dissolution of the lipid intercellular matrix occurs [22]. This is known as the ethanolic effect presented schematically on Figure 3A. Under the influence of ethanol, lipid membranes become more plastic, including the ethosome’s own lipid membranes. This improves the ethosome’s ability to release the drug in deeper layers of the epidermis. This is the ethosomal effect depicted on Figure 3B [23].

By locally administering drugs enclosed in ethosomes, the first-pass effect in the liver and the degradation of the drug in the gastrointestinal tract are avoided. In addition, the toxicity of the drug and the amount and severity of the side effects are reduced. Reducing the frequency of drug administration may have an influence on the effectiveness of therapy and patient compliance [24].

## 3. Methods of Preparation

**Figure 4:**
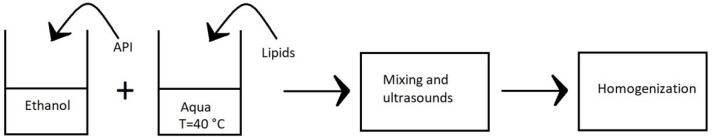
The hot method. API—Active Pharmaceutical Ingredient.

Three methods are used to obtain ethosomes. The first is the hot method visualized on Figure 4. A small amount of the medical substance is dissolved in ethanol or a mixture of ethanol and glycol. Then, it is added to phospholipids dispersed in water at 40 °C. Everything is mixed using a magnetic stirrer for 5 min. The prepared mixture is subjected to ultrasound treatment at 4 °C for 3 cycles of 5 min. Between cycles, there is a 5 min time interval. In order to obtain particles of a few nanometers in size, the drug form is homogenized in a high-pressure homogenizer or by using a sonification or extrusion process [25,26].

A variation of the hot method used by Touitou in studies with minoxidil (2,4-Pyrimidinediamine,6-(1-piperidinyl)-,3-oxide) involves the heating of a soybean lecithin suspension and water to 60–70 °C. Then, the dispersion system is cooled in an ice bath. With continuous stirring for 30 min, a high-concentration solution of ethanol and propylene glycol is added. The preparation is then homogenized [3].

**Figure 5:**
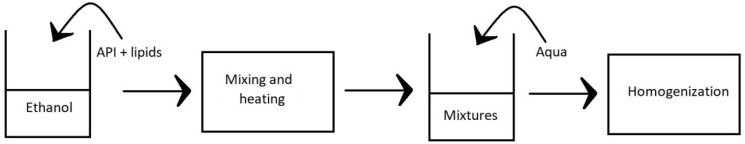
The cold method.

The second method of obtaining the ethosomes is the cold method—Figure 5. This is one of the most popular methods for obtaining the ethosomal forms of the drug. Phospholipids, the drug, and other lipophilic ingredients are dissolved in ethanol at room temperature. Then, the mixture is stirred. The polyols are added during the stirring process. Next, the drug form is heated to 30 °C in a water bath. The heated water (30 °C) is added to the obtained mixture while it is being stirred. A suitable particle size is obtained by sonification or extrusion. The final form of the drug is stored in a cool place [27,28].

**Figure 6:**
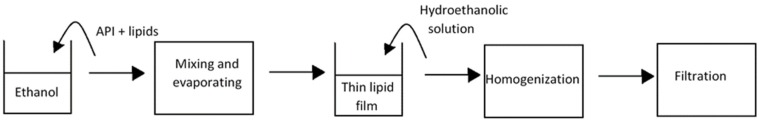
The classic method of mechanical dispersion.

A different method of mechanical dispersion (Figure 6), known as the classic method, has been reported by Chen et al. [29] whereby they dissolved lecithin and curcumin in a glass bottle containing ethanol. The method was enhanced by combined thin-film hydration and ultrasound homogenization. The resulting product was mixed using a magnetic stirrer. The mixture was then poured into a round-bottom flask. A thin film was created using a rotary evaporator. They then hydrated the resulting film to obtain ethosomes, however, the remaining content of ethanol in sonificated ethosomes was not evaluated. The ethosomes were homogenized for 5 min by sonification. Next, the ethosomes were filtered off using a 0.22 µm filter.

## 4. Types of Drugs Encapsulated in Ethosomes

Research on ethosomes was initially conducted by Touitou [23]. The objective of this field of study is to obtain increased penetration of active substances and to prevent skin irritation. New preparations should be safe and comfortable to use for the patient [30]. The structure of the ethosome allows for increased penetration of substances poorly soluble in water.

In Table 1, the therapeutic groups of drugs encapsulated in ethosomes are listed.

The variability of the APIs applied in ethosomal form suggests the possibility for the use of ethosomes in parenteral preparations, however, the ethanolic content of the formulation is a serious restriction.

## 5. Anti-Viral Drugs

The research conducted by Zhou et al. [34] led to a composition containing aciclovir. Acyclovir encapsulated in ethosomes has repeatedly increased the penetration capacity through the epidermal layers. The content of the active substance in the epidermal layer was tested and compared for acyclovir and the acyclovir prodrug. The prodrug was tested in an ethosomal form and in the form of an aqueous–alcoholic solution. In addition, the ethosomal form of acyclovir was also used for comparison. The experiment was conducted for 24 h. It was shown that the content of the ethosomal acyclovir prodrug was 62.289 µg/cm^2^ and was 5.3 times higher than the concentration of the acyclovir prodrug in the form of an aqueous–alcoholic solution. In addition, it was also 3.43 times higher than the concentration of the ethosomal form of acyclovir [34]. Studies have been conducted exploring the use of ethosomes that allow an increased penetration of other antiviral drugs such as zidovudine [35] and lamivudine [36]. The percutaneous penetration of zidovudine in the ethosomal form and its penetration in the liposomal form were compared. Higher skin permeability for ethosomes was demonstrated by using fluorescence microscopy using rhodamine 123 as a fluorescent probe. It has been shown that the zidovudine penetration rate through the skin for the ethosomal form is 78.5 +/− 2.5 µg/cm^2^/h compared to 5.2 +/− µg/cm^2^/h for a water–ethanol solution and 7.2 +/− 0.6 µg/cm^2^/h for an ethanol solution [35]. Transmission electron microscopy and scanning electron microscopy have been employed with the aim of determining the effects of ethosomes on the skin structure. Placing the active ingredient in an ethanolic solution inside ethosomes increased the penetration of the drug through the skin of rats from 2.8 +/− 0.2 µg/cm^2^/h to 68.4 +/− 3.5 µg/cm^2^/h compared with lamivudine liposomal solution [36]. In addition, indinavir has also been studied. The use of the ethosomal form of drug reduces the severity and frequency of side effects [28].

The content of ethanol may improve the stability of formulations. The literature suggests that the combination of phospholipids and a high concentration of ethanol in formulations can demonstrate synergistic effects. It may have an influence on higher penetration in the skin lipid bilayers giving a deeper distribution.

## 6. Antifungal Drugs

The literature describes the positive influence of the content of the ethanol in antifungal topical drugs. In the treatment of fungal diseases of the skin and its appendages, achieving a sufficient concentration of the active substance at the site of infection is a problem which is difficult to develop in a drug form. The literature describes attempts to create a fluconazole-containing drug used in the treatment of skin and mucosal candidiasis. An ethosomal form of the drug prolongs the time that the drug remains in the epidermal layer, accelerating healing. It reduces the frequency of side effects and the duration of therapy [25]. Franz cells were used in diffusion studies. The effective diffusion surface of the mouse skin was 22.51 cm^2^. The percentage of the drug released from the drug form in ethosomes, liposomes, and fluconazole hydroethanolic solution was 89.57%, 49.48%, and 32.64%, respectively. In addition, it was observed that with increasing ethanol concentration, the percentage of drug diffusion increases. This phenomenon occurs as the ethanol content increases up to 30%. Increasing the ethanol content above 30% results in the inhibition of the diffusion of the active substance [25]. Clotrimazole is also often used in the treatment of candidiasis. Maheshwari et al. demonstrated that drug placement in ethosomes increases its solubility and the ability to remain in the lipid bilayer of the skin [37]. A formulation containing clotrimazole in the ethosomal form was characterized by an increased penetration through the epidermal layer (56.25 +/− 5.49 µg/cm^2^/h) and a reduced time lag (54 min) compared to the ultra-deformable liposomes (diffusion flow 50.16 +/− 3.84 µg/cm^2^/h, lag time 60 min). The inhibition zones of growth against *Candida albicans* were also tested in vitro. The ethosomal form of the drug was characterized by a larger zone of growth inhibition (34.6 +/− 0.57 mm) compared to classic liposomes (29.6 +/− 0.57 mm). The use of creamy market preparations has been shown to be the least favorable as regards to growth inhibition zones. In this case, the growth inhibition zone was only 19.00 +/− 1.00 mm [37]. The active substance used to treat infections caused by fungi of the genus *Microsporum* and *Trichophyton* is griseofulvin. Pharmacodynamics of this drug encapsulated in ethosomes were performed by Aggarwal and Goindi. The researchers determined the effect of different proportions of Phospholipon^®^ 90G and ethanol on the degree of drug entrapment in ethosomes and the size of the vesicles. They established a composition that was characterized by the highest degree of entrapment of the therapeutic substance within the ethosomes. The selected formulation contained 30% ethanol and 200 mg of Phospholipon^®^ 90G. The optimal form of this drug was subjected to further testing on mice. The level of penetration through the epidermis and drug retention in the skin of mice was examined by administering ethosomal gel three times daily for 3 days to the test group. The control group was subjected to the same treatment with physiological saline. After the application of the ethosomal gel with griseofulvin, edema, the infiltration of inflammatory cells, erythema, scaling of skin or dryness of skin in mice was not observed. Ex vivo studies showed that the drug penetration for the optimal formulation was 70.77 +/− 0.83%, that of the aqueous suspension was 9.68 +/− 0.86%, that of the creamy form was 25.58 +/− 0.61%, that of the water–alcohol gel was 39.37 +/− 0.79%, and that of liposomal dispersion was 52.01 +/− 0.73%. The rate of drug retention in the skin grew in the same order as the order of these degrees of penetration. The skin of mice was treated with Rhodamine B-containing ethosomes and the degree of penetration was observed after 4 h under a fluorescence microscope. The growth inhibition zones for dermatophytes of the genus *Microphyton* and *Trychophyton* were also compared. The inhibition zones for the griseofulvin liposomes were smaller than the zones for the ethosomal form. This was associated with a greater degree of penetration of the active substance from the ethosome than that from the liposome [38]. Zhang et al. investigated the ability of the skin to penetrate ethosomes and transferosomes with terbinafine hydrochloride. The characteristics of the obtained forms of the drug were developed on the basis of the average size of bubbles, zeta potential, and the degree of drug durability. The obtained results indicate that the highest content of active substance in the skin, the degree of drug retention in the epidermis, and the skin diffusion rate were characterized by binary ethosomes containing ethanol and propylene glycol in a weight ratio of 7:3. Binary ethosomes had the smallest bubble diameter and the highest stability [39]. Increasing the effectiveness of polyene antibiotic antifungal–amphotericin B was achieved by using the drug form with ethosomes. Liposomal, ethosomal, and transferosomal dispersions, as well as gels with liposomes, ethosomes, and transferosomes, were compared. The amount of the drug retained in the skin was tested in vitro in Franz’s cells in the skin of rats. A significant increase in penetration was observed for transferosomal dispersions. The amount of the drug retained in the skin was also highest. For transferosomes in the form of a gel it was 81 µg, for transferosomal dispersion it was 77 µg. For comparison, it was 64 µg for the ethosomal gel and only 45 µg for the liposomal gel. The scattering was characterized by lower values of the retained drug in the skin compared with the corresponding gel forms. For the ethosomal dispersion, the amount of drug retained in the skin was 56 µg, and for liposomal dispersion, it was 35 µg. The amount of the retained drug was tested 24 h after application. Growth inhibition zones for the *Trychophyton* fungi were also examined. The size of the growth inhibition zone was tested three days after application. Maximum inhibition was observed for transferosomes, which is explained by a higher degree of release of the active substance from the lipophilic carrier [40].

One cannot exclude the synergistic effect of the ethanol contained in ethosomes with antiviral, antifungal, and antibacterial drugs.

## 7. The Effects of Drugs on Cellular Metabolism

The 5α-reductase inhibitor finasteride used in the treatment of mild prostatic hyperplasia and in androgenic alopecia was enclosed in ethosomes. The studies were conducted in vitro using ethosomal drug carriers. Transdermal penetration of finasteride in ethosomal form was 7.4, 3.2, and 2.6 times higher than that of an aqueous solution, liposomes, and hydroethanolic solution, respectively. Accumulation of finasteride in the skin from the ethosomal formulation was 18.2 +/− 1.8 µg/cm^2^ in comparison to 2.8 +/− 1.3 µg/cm^2^ for the liposomal form. Rao et al. studied the distribution of finasteride in the various layers of the skin. Aqueous, hydroalcoholic solution and liposomes were located mainly in the epidermal layer. The ethosomal form of the drug was located in the dermis [48]. Minoxidil is also the drug used enclosed in ethosomes for the treatment of hair loss [52,53]. Hong-You et al. have shown by HPLC that the closing efficiency of minoxidil in ethosomes is from 61.1 to 63.3% [53].

The flux through the skin of the active substance results from the higher solubility in the presence of ethanol.

## 8. Antioxidants and Flavonoids

Chiu et al. compared the gelling process of ethosomes containing α-tocopherol acetate with hollow ethosomes using water-soluble polymers. The physical properties of empty ethosomes and ethosomes containing α-tocopherol acetate were compared. It was shown that the best yielding capacity was characterized by ethosomes containing 20% ethanol (93 +/− 6.3%). A two-fold increase in ethanol concentration resulted in a yield decrease to 16 +/− 5.6%. It has been shown that increasing the alcohol concentration reduces the stiffness of the lipid particles [43]. Many other medicinal substances are enclosed in the ethosome including quercetin [44] and rutin [45]. In the research regarding quercetin, the optimal formulation was demonstrated to be 2% phosphatidylcholine and a 20% ethanol solution. The stability of ethosomes also depended on the content of quercetin. The most stable form was a drug containing 0.04% quercetin. It is important to note that increasing the ethanol content to 40% and phosphatidylcholine to 5% resulted in an instability of the obtained compositions. It was observed that the efficiency of closing ethosomes increased with the increasing concentration of quercetin. In addition, the ability of ethanolic solution and liposomes to penetrate ethosomes was compared. The amount of the drug in the stratum corneum of mouse skin was as follows: for ethosomes 18.8 +/− 0.6 µg/cm^2^; for the ethanolic solution 15.1 +/− 1.7 µg/cm^2^; for liposomes 8.6 +/− 1.9 µg/cm^2^; and for distilled water 0.1 +/− 0.02 µg/cm^2^ [44]. Park et al. showed that rutin-loaded ethosomes (0.005–0.03% concentration) were stable during a four-week observation. A 0.03% solution was used for in vitro skin penetration tests. It was shown that ethosomal drug forms are the most effective carriers for rutin. The total skin penetration rate of rutin was 61.3% for the ethosomes; 44.16% for ethanol solution; 37.80% for liposomes; and 18.31% for distilled water. A comparison of the liposome containing 0.03% rutin with ethosomes showed that the ethosomes were characterized by a larger particle size (190.20 +/− 14.57 nm) but also by a greater flexibility (9.75 +/− 0.3). For comparison, the following values were obtained for liposomes—particle size 92.4 +/− 2.28 nm, flexibility 3.74 +/− 0.29 [45].

The content of the ethanol may improve the elasticity of the particles in formulations. Flavonoids have poor water solubility. It may be improved through the addition of ethanol.

## 9. Antineoplastic Drugs and Immunomodulatory Drugs

Anti-cancer drugs are an important therapeutic group of drugs. Chen et al. conducted research on curcumin showing immunomodulatory, antioxidant, and antineoplastic activity. The obtained results of the minimal inhibitory concentration (MIC) and minimal bactericidal concentration (MBC) tests showed that curcumin used in the form of ethosomes has a much higher penetration rate and ability to penetrate through the model membrane. The best retention performance was found in the 45% (*v*/*v*) ethanol and 2% (*w*/*v*) phospholipids prepared ethosomes. The ethosomes were of a small size (50.32 +/− 5.13 nm) and had a good distribution capacity [24]. Tamoxifen in the form of ethosomes was characterized by an increased penetration through the cellophane membrane and human skin. The comparison carried out by Sarwa et al. concerned the penetration profile of ethosomes, liposomes, and the hydroethanolic solution through the skin. The obtained results showed that within 24 h of administration, the ethosomes provided more than 90% of the active substance, liposomes 39.04%, and the water and ethanol solution only 36.55% [42]. An extract from *Tripterygium wildfordii* is used in Chinese medicine. These extracts have anti-inflammatory, antibacterial, and immunoregulatory properties, as well as antineoplastic activity. The use of these extracts over long periods causes serious side effects. The use of ethosomal drug carriers allowed for a reduction in MIC and MBC which resulted in a reduction in the frequency of the side effects. In studies of triptolide, the active substance isolated from *Tripterygium wildfordii*, there was an inverse relationship between the diameter of ethosome particles and ethanol concentration. The size of ethosomal beads increased as the concentration of phospholipids increased. The smaller particle size, the higher the degree of binding of the active substance, with a good distribution being characterized by the ethosomes containing 2% (*w*/*v*) lecithin and 45% ethanol (*v*/*v*) [54].

Ethosomes are characterized by good deformability, satisfactory permeability, and high-entrapment efficiency. They improve permeability for lipophilic drugs.

## 10. Antibacterial Drugs

Ethosomes containing erythromycin were tested in vitro and in vivo in mice. The studies demonstrated that the topical use of erythromycin in ethosomal form reduces the MIC by half for *Staphylococcus aureus* compared to the use of an aqueous–ethanol solution. Histopathological observations of the skin of mice have shown that ethosomal carriers penetrate efficiently into the deeper layers of the skin, which makes it possible to fight staphylococcal infections in dermis [31]. Glujoy et al. have shown that ethosome-encapsulated drugs are characterized by a greater deposition of the active substance in the deeper layers of skin, greater efficacy, and increased the safety of pharmacotherapy compared to liposome and niosome systems [55]. The ethosomes may positively interplay with the above-mentioned drugs, and the ethanol may enhance the antibacterial effect of the antibiotic.

## 11. Methods of Testing

To determine the concentration and antibacterial properties of clotrimazole, Liu et al. performed an experiment on rats. Rat skin penetration was investigated in Franz cells. The concentration of clotrimazole was determined by HPLC. Antibacterial and antifungal activity was assessed against *Candida albicans*, *Escherichia coli*, *Stapholococcus aureus*, and *Pseudomonas aeruginosa*. The MIC, after application of the ethosomes, was reduced by 50% when compared with that of the drug in liposome form [56]. The efficacy of minoxidil in the form of ethosomes was examined on the skin of hairless abdominal mice. The amount of transport through the skin was tested in Franz cells under specific conditions—temperature 37 °C and medium volume 4–8 mL. The concentration of the substance in the samples was examined by HPLC [3]. Tamoxifen citrate is administered long-term in the treatment of many hormonal dermatological disorders. Ethosomal delivery is an alternative to oral and parenteral administration. Oral administration is characterized by the high enzymatic degradation of the composition. In addition, tamoxifen citrate is poorly soluble in water. The penetration testing of the active substance was carried out using a cellophane membrane and the skin from human corpses. The obtained results were compared with the penetration of liposomes. The results showed that ethosomes allowed for a penetration of more than 90% of the drug, while liposomes, only approximately 39% [42].

## 12. Stability

The stability of an ethosome-containing drug can be determined by measuring the particles and drug structure over time. The size of the particles can be evaluated using dynamic light scattering (DLS) measurements [57]. Changes in the structure of ethosomes can be evaluated using a transmission electron microscope. The use of DLS measurements together with photon correlational spectroscopy allows for the evaluation of the zeta potential of particles and its relation to the size of ethosomal balls [58]. Electrokinetic properties play an important role in understanding the mechanisms at work at the interface between the disperse phase and the diffusing phase [59]. The zeta potential (ζ) is the electrical potential at the interface [60]. The presence of potential is related to the distribution of ions and counterions on the surface of particles and in the region adjacent thereto. Inductive load formation depends on the pH and ionic strength of the dispersing phase. The zeta potential can be one of the indicators of the physical stability of the control system in semisolid systems because it describes the level of attraction and repulsion between individual particles [61]. Determination of the zeta potential is related to the electrophoretic mobility, *u_E_*, which is defined as a ratio of the end speed *v* [cm/s] to the electric field intensity *E* [V/cm].

(2) $$u_{E}=\frac{\upsilon }{E}$$

Determination of the zeta potential according to the Dukhin and Derjaguin procedures involves multiple solutions of the following equation:

(3) $$E=\frac{3\bar{\zeta }}{2}-6\{\frac{\bar{\zeta }}{2}-\frac{\ln2}{z}\left[1-\exp\left(-z\bar{\zeta }\right)\right]\}\times {\left\{2+\frac{\kappa a}{M}\exp\left(\frac{z\bar{\zeta }}{2}\right)\right\}}^{-1}$$

where: $E=\frac{3\eta eu_{E}}{2\varepsilon_{r}kT}$, and: $M=\frac{2\varepsilon_{r}N_{Av}kT}{\eta \Lambda }$, $\bar{\zeta }$ is the zeta potential, *η* is the medium viscosity, *ε_r_* is the dielectric constant, *k* is Boltzman’s constant, *N_Av_* is the Avogadro number, Λ is the specific conductance of the solution, *u_E_* is the electrophoretic mobility, *T* is the temperature, *a* is the radius of particles, and *κ* is the Debye length [62].

Lipid membranes interact with each other. There are phenomena of adsorption, aggregation, and Fusion. In this way, the lipid bilayers of the ethosomes react with the lipid membrane of the epidermal cells [63]. The Stern equation, in combination with the Langmuir’s adsorption isotherm, Boltzman’s relations, and Graham’s equation enabled the development of a theoretical description of the adsorption of charged particles on the surface. The described equation was used, amongst others, in studies on the adsorption of divalent cations on the surface of biological membranes containing phosphatidylserine [64]. Dispersive systems also have surface tension and osmotic pressure. Söderlund et al. compared the effect of both phenomena on the interfacial hydration of the lipid bilayer [65]. The properties of the liposome-dispersing medium influence the stability of colloidal systems. The dispersion of these particles in a hydrophilic matrix with a certain viscosity, density, and osmotic pressure is of great importance. Williams et al. studied the dependence of osmotic pressure on the density of the colloidal system [66]. Interactions in gel drug forms are described by many theories, such as that of Flory and Rehner. It is a combination of two statistical models: the Flory–Huggins model, which describes the mixing of a hydrophilic polymer and a solvent; and the Gaussian-chain model, which describes the flexibility of a gelling network [67]. The Flory–Huggins model can be used to describe the branching of polymers used as well as the limitations in the polymerization process. It is a tool enabling the characterization of various associative systems. The Flory–Huggins model is based on the assumption that a single molecule polymerizes and depolymerizes according to a dynamic equation:

(4) $$\Delta f_{p}=\Delta h_{p}-T\Delta S_{p}$$

where Δ*h_p_* is the enthalpy of the polymerization reaction, Δ*S_p_* is the entropy of the polymerization reaction, and *T* is the temperature [68], and these issues are further developed for liposome systems [69,70], evaluated in various practical applications [71].

## 13. Conclusions

Ethosomes show important practical advantages compared to classic liposomes. In particular, their usage improves the bioavailability of compositions that are both easily and poorly soluble in water. The advantages of using liposomes as a transdermal patch include improved hydration of the outer layer of the epidermis, a potential increase in absorption, a reduction in side effects while improving patient compliance. There are several theoretical methods useful for the production of liposomes with an alcohol fraction, but in practice, only some of them are used in the pharmaceutical industry, in particular, in the production of cosmetic creams, anti-hair loss preparations, and topical antiviral preparations. It is necessary to conduct research with regard to real pharmaceutical systems using advanced physicochemical techniques. The literature describes many examples of ethosomes being more effective than other encapsulating forms. They increase the solubility of pharmaceutical ingredients and permeability through the skin in comparison of hydroethanolic solutions, liposomes, and aqueous solutions. However, the positive activity of ethanol contained in ethosomes may be accompanied by a risk of denaturation of some proteinic components, such as bioderivatives applied in ethosomes, as well as potentially causing irritation of the skin and mucous surface.

## Figures and Tables

**Table 1:** The therapeutic groups of drugs encapsulated in ethosomes.

Therapeutic Group of Drugs	Type of Active Pharmaceutical Ingredient (API)	Literature
Antibacterial	Erythromycin	[31]
Anti-inflammatory	Ammonium glycyrrhizinate	[32]
	Diclofenac	[33]
Anti-viral	Acyclovir	[34]
	Zidovudine	[35]
	Lamivudine	[36]
	Indinavir	[28]
Anti-fungal	Fluconazole	[25]
	Clotrimazolum	[37]
	Griseofulvin	[38]
	Terbinafinum	[39]
	Amphotericin-B	[40]
Antineoplastic	Curcumin	[41]
	Tamoxifen	[42]
Antioxidants and flavonoids	α-tocopherol acetate	[43]
	Quercetin	[44]
	Rutin	[45]
Hormones	Testosterone	[46]
Hypnotic	Melatonin	[47]
5α-reductase inhibitor	Finasteridum	[48]
Steroids	Clobetazol	[49]
Tertiary amine	Tolterodine tartrate	[50]
Vaccines	Hepatitis B Antigen	[51]
